# A narrative review of glucagon-like peptide-1 receptor agonists prior to deep sedation or general anesthesia

**DOI:** 10.1186/s44158-025-00237-y

**Published:** 2025-03-28

**Authors:** Luigi Vetrugno, Cristian Deana, Andrea Da Porto, Enrico Boero, Valentina Bellini, Daniele Guerino Biasucci, Elena Giovanna Bignami

**Affiliations:** 1 Department of Emergency, Health Integrated Agency of Friuli Centrale, Tolmezzo, Italy; 2 Department of Medical, Oral and Biotechnological Sciences, University of Chieti-Pescara, Chieti, Italy; 3 Department of Anesthesia and Intensive Care, Health Integrated Agency of Friuli Centrale, Udine, Italy; 4 Diabetes and Metabolism Unit, Department of Internal Medicine, Health Integrated Agency of Friuli Centrale, Udine, Italy; 5 Anesthesia and Intensive Care Unit, San Giovanni Bosco Hospital, Turin, Italy; 6 Critical Care and Pain Medicine Division, Department of Medicine and Surgery, University of Parma, Parma, Italy; 7 Department of Clinical Science and Translational Medicine, Tor Vergata’ University of Rome, Rome, Italy

**Keywords:** GLP-1 receptor agonists, Anesthesia, Perioperative period, Fasting, Ultrasound

## Abstract

Glucagon-like peptide-1 receptor agonists are a class of drugs that mimic a natural incretin hormone released by the intestine after meals, and they are well-suited for treating type 2 diabetes. Glucagon-like peptide-1 receptor agonists also lead to satiety and appetite reduction through action on the brain’s appetite regulation centers, leading to weight loss in obese patients. However, because glucagon-like peptide-1 receptor agonists work to slow gastric emptying, a safety concern has been raised in patients undergoing deep sedation or general anesthesia regarding gastric aspiration, and considering their long half-life in the blood, they are difficult to manage in the perioperative period. The purpose of this review is (i) to explore the present knowledge about the risk of aspiration before anesthesia; (ii) to describe the method for evaluating the presence of liquid and food in the stomach before surgery; and (iii) to balance the actual warning with the opportunity for future discovery about their benefits.

**Supplementary Information:**

The online version contains supplementary material available at 10.1186/s44158-025-00237-y.

## Background

Nowadays, metabolic disorders are increasing rapidly worldwide, and incretin-based therapies have gained a pivotal role in the management of type 2 diabetes and obesity [1, 2]. The pharmacology of drugs such as Glucagon-like peptide-1 receptor agonists (GLP-1 RAs) and dual incretin receptor agonists is mainly characterized by two mechanisms of action. Firstly, by restoring “the incretin effect” whereby oral nutrients elicit insulin release and glucagon suppression [3]; these drugs exert a potent, safe, and glucose-dependent hypoglycemic effect. Secondly, these molecules induce substantial weight loss because of their dose-dependent central inhibition of energy intake and mitigation of food cravings [4]. Moreover, in light of the growing evidence demonstrating the benefits of incretin-based therapies on both metabolic health and cardiovascular diseases, regulatory agencies such as the Food Drug Administration (FDA) and the European Medicines Agency (EMA) have approved their use for type 2 diabetes, reduction of cardiovascular risk and for chronic weight management in patients with morbid obesity [5–8]. These new treatment possibilities for clinicians, coupled with an increase in industry marketing and patients’ personal needs, have led to an explosion of prescriptions for these drugs especially in Western countries [9].

From the “anesthesiologist” point of view, the growing number of patients treated with incretin-based therapies undergoing deep sedation and general anesthesia uncover the possible dark side of these drugs such as revealed by many case reports documenting the association between general anesthesia and gastric aspiration of a large amount of gastric content (including undigested food particles) in patients taking GLP1-RAs during the peri-operative period [10–12]. The potential mechanisms underlying the increased risk of gastric aspiration are not fully understood, and nowadays it is explained mainly by delayed gastric emptying and inhibition of gastrointestinal motor function that GLP-1-RAs exert through both central motor nuclei and vagal afferents [13, 14]. Moreover, newer GLP-1 RAs and the long half-life of dual agonists (up to 7 days for Semaglutide) give rise to longer preoperative fasting period requirements and concerns of perioperative management of patients, even in case of treatment interruption [15–17].

### Aims of the narrative review

The purpose of this review is threefold: (i) to explore current knowledge regarding the risk of aspiration prior to anesthesia; (ii) to describe the methods used to assess the presence of liquids and food in the stomach before surgery; and (iii) to weigh the current warnings about GLP-1 RAs against the potential for future discoveries regarding their benefits”.

### Analysis of specific topic

This literature review was conducted following the PICO Framework, with details available in Supplementary material 1. This literature review was conducted following the PICO Framework, with details available in Supplementary material 1.

### Does gastric residual content really increase in patients who take GLP-1 RAs?

To date, mechanistic studies that evaluate gastric emptying in patients chronically exposed to pharmacological doses of GLP-RAs have produced extremely variable and often poor-quality results. Many studies have proved that gastric emptying rates consistently show inter-individual variation in the general population [18]. In addition, prolonged exposure to pharmacologic doses of long-acting GLP-1 RAs induces tachyphylaxis for the effects on gastric emptying, and that is why nausea vanishes quickly after the beginning of treatment in most patients [19]. The activation of the area postrema during the peak levels of GLP-1 RAs may provide an explanation for gastrointestinal symptoms. If nausea arises when GLP-1 RA plasma concentrations are at their peak, then continuous exposure to GLP-1 RAs may lead to tachyphylaxis—a reduced response to the drug over time—resulting in fewer gastrointestinal symptoms after long-term use [20]. On the other hand, much evidence from large retrospective cohorts with endoscopic evaluation of gastric content has proven the association between incretin-based therapy exposure and increased retained gastric content [21].

Silveira et al. recently studied the risk of broncho-aspiration in 886 patients who underwent esophagogastroduodenoscopies (EGDS) under general anesthesia. Of these, 404 patients were taking GLP-1 RAs 30 days prior to the procedure, while 371 were not. The study showed an increase in residual gastric content in 27 patients (6.7%), 8 (24.2%) in the Semaglutide, and 19 (5.1%) in the non-Semaglutide group (*p* < 0.001). The presence of nausea/vomiting, dyspepsia, and abdominal distension were also associated with increased residual gastric content in patients taking Semaglutide with a propensity-weighted analysis of 3.56 (95% CI 2.2–5.78)] [22]. Santos et al. did a second study aimed at defining the time interval at which residual gastric content becomes comparable between the Semaglutide and non-Semaglutide groups without symptoms. The study included 1094 patients: Semaglutide = 123; non-Semaglutide = 971). Increased residual gastric content was observed in 56 (5.12%), 25 (20.33%) in the Semaglutide and 31 (3.19%) in the non-Semaglutide group (*p* < 0.001). Following weighted analysis, the presence of nausea/vomiting, dyspepsia, and/or bloating/abdominal distension disappear only after a time interval of ≥ 14 days without association in residual gastric content between the two groups [OR = 0.77 (95%CI 0.22–2.01)] [23]. However, both these studies were retrospective. Kobori T et al. studied 1128 individuals with diabetes who had EGDS and carried out a one-to-one nearest neighbor propensity score matching analysis between diabetes patients treated with and without GLP-RAs. They found that in patients undergoing EGDS, the proportion of solid gastric residue was nearly tenfold higher in the GLP-1-RA group (5.4%) compared to controls (0.49%) (*p*.0.004) [24].

Yeo et al., in a retrospective cohort analysis utilizing the TriNetXdataset and revealed that among over 20,999 users of GLP-1 RAs, both the type of procedure and the method of sedation significantly increase the risk of aspiration. Specifically, upper gastrointestinal procedures are associated with a higher risk, and the use of propofol further exacerbates this risk. These findings underline the critical importance for anesthesiologists to carefully consider their choices regarding procedures and sedation methods in order to effectively reduce these risks [25]. Based on these studies, anesthesiologists may empirically assume that the risk of increased residual gastric content in fasted patients on Semaglutide/GLP-1-RAs are 5–10 times higher than in fasted patients not on Semaglutide/GLP-1-RAs. This means that in patients taking GLP-1 RAs, the preprocedural fasting time endorsed by current guidelines (a minimum of 2 h for clear fluids, 6 h for a light meal, and 8 h for a meal that includes fried or fatty food) may be inadequate and may increase the risk of aspiration under anesthesia [26, 27].

### Can we measure gastric transit time or residual gastric content in patients taking GLP-1 RAs?

Currently, there are four methods used to measure the time it takes for food to transition through the stomach, one of which involves examining the presence of residual gastric content. Of the first 3, nuclear scintigraphy is a direct measurement and the gold standard technique [28] followed by the Gastric Emptying Breath Test (GEBT), a non-invasive procedure that uses a test meal containing ^13^C-Spirulina platensis [29]. This test allows for accurate measurement of gastric emptying by analyzing the exhalation of ^13^CO2, providing a non-radioactive alternative to scintigraphy. The other two methods are the acetaminophen absorption test and transcutaneous fluorescence spectroscopy [30, 31]. The acetaminophen absorption test is based on the principle that acetaminophen is absorbed in the duodenum rather than the stomach. Therefore, measuring its appearance in the plasma after concurrent intake of food can provide an estimate of gastric emptying time. However, this method has several limitations, most notably the requirement to collect 10–20 blood samples. Recently, researchers have explored transcutaneous fluorescence spectroscopy for non-invasive measurement of liquids and meals using fluorescein as a contrast agent. A wearable probe detects the fluorescence signal in the bloodstream as the agent passes from the gut into the blood. While some experiments have been conducted in this area, the method has not yet been validated in patients taking GLP-1 RAs. Despite being the gold standard, nuclear scintigraphy is not suitable for everyday clinical practice due to its invasive nature, radiation exposure, and high costs and the GEBT has been endorsed by consensus statements from the American and European Neuro-gastroenterology and Motility Societies [32]. Gastric ultrasound has emerged as the most highly recommended technique by various anesthesiology societies for assessing the presence of residual gastric content [33]. This assessment helps estimate a patient’s risk of aspiration. Additionally, gastric ultrasound allows for real-time evaluation of gastric volume at the bedside, which supports personalized risk assessment and management for each patient” (Table 1).

**Table 1:** SWOT analysis of gastric ultrasound in clinical practice

Strengths	Weaknesses
Easy and fast to use	Challenging in obese patients
Feasible and repeatable at bedside	Patient factors limiting feasibility (e.g., abdominal scarring, excessive bowel gas, or specific acute abdominal pathologies)
Radiation-free	Logistical constraints (e.g., time constraints in emergency settings or limited access to ultrasound equipment)
Non-invasive and safe	Operator dependency
Opportunities	Threats
Preoperative assessment in high-risk patients (e.g., delayed gastric emptying, diabetes, or opioid use)	Variability in operator skill and experience
Emergency settings (e.g., trauma or critically ill patients requiring rapid gastric content assessment)	Limited awareness or adoption in some clinical environments
Pediatric patients with uncertain fasting status	Cost for training and equipment

### How to perform gastric ultrasound and most relevant studies

**Fig. 1:**
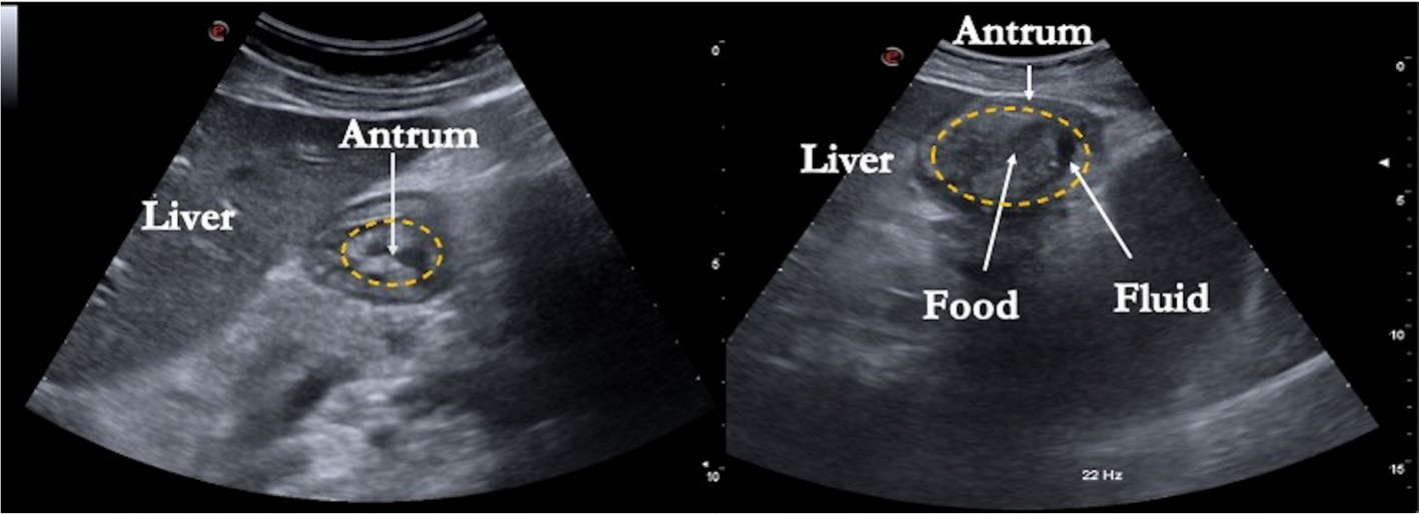
Gastric ultrasound evaluation

Gastric ultrasounds are usually performed with the patient in the supine and the right lateral decubitus position with a convex probe (2–5 MHz) positioned in the epigastric region. Solid content appears hyperechoic, while gastric secretions appear hypoechoic, providing both qualitative and quantitative information about gastric content (Fig. 1). In a randomized study on 80 healthy volunteers, gastric ultrasound had a high diagnostic accuracy with a sensitivity of 1.0 (95% confidence interval [CI], 0.925–1.0), a specificity of 0.975 (95% CI, 10.33–∞), a positive predictive value of 0.976 (95% CI, 0.878–1.0), and a negative predictive value of 1.0 (95% CI, 0.92–1.0) [34]. In a recent well-performed experiment study, Van de Putte P. used gastric ultrasound to examine the gastric content in 538 patients undergoing elective surgery under general anesthesia who had respected institutional fasting guidelines. Of these patients, 6.2% presented with a full stomach. Nine of these (1.7%) had solid content, and 23 (4.5%) had clear fluid > 1.5 ml kg^1^. An empty stomach was documented in 480 (89.8%) patients [26]. This study suggests that a small proportion of elective surgical patients, 6.2%, may present with a full stomach despite the recommended duration of fasting. Another important finding in this study was that gastric ultrasound was inconclusive in 20 patients (5.0%). It should also be underscored that obesity and poor ultrasound windows are both enemies of ultrasound. Knowing these limitations, Sherwin M. et al. used gastric ultrasound in non-obese volunteers taking Semaglutide. The authors found that 70% of the patients in the supine position taking Semaglutide, and 10% in the control group, showed residual solid gastric content following an overnight fast and 2 h after taking clear liquids [35]. This finding could have significant implications because it suggests a higher risk of aspiration of 3.5 (95% CI 1.26 to 9.65) during anesthesia in patients taking Semaglutide compared to the control group. However, the sample was small and made up of volunteers. Again, gastric ultrasound was used in a recent prospective observational study, which included 220 patients. Of these, 107 were in the Semaglutide group and 113 in the non-Semaglutide group, with residual gastric content found in 43 of 107 patients (40%) taking Semaglutide vs. 3 of 113 (3%) in the non-Semaglutide group (*p* < 0.001), showing that a preoperative Semaglutide use within 10 days of elective surgical procedures was independently associated with increased risk of residual gastric content evaluated with gastric ultrasound [35]. The prevalence of increased residual gastric content with gastric ultrasound was shown in a prospective study by Sen et al. in 124 patients. Of these, RGC was present in 56% of patients with GLP-1 RAs compared with 19% of patients without GLP-1 RAs. After adjustment, confounding GLP-1 RAs were associated with a 30.5% (95% CI − 9.9–51.2%) higher prevalence of increased residual gastric content [36].

In contrast, a recent small study showed that continuation of GLP-1 RAs at endoscopy in a case series of 57 adult patients undergoing endoscopic sleeve gastroplasty underwent ESG without stopping GLP1-RAs, which included Semaglutide (45.6%), Liraglutide (19.3%), Dulaglutide (22.8%), and Tirzepatide (12.3%). During intubation, endoscopy, and recovery, there were no retained gastric solids, pulmonary aspiration, gastroesophageal regurgitation, or hypoxia [37], which raises doubt about the previous studies and invites consideration that other patients may have had other clinical conditions such as bowel dysmotility, gastroparesis, or Parkinson’s disease [38, 39].

### How confusing is the situation, given the different guidelines worldwide?

Following the literature reporting the association between case reports and aspiration under anesthesia and the study with gastric ultrasound highlighting the risk of increased gastric content in patients taking GLP-1 RAs [10, 11], in June 2023, the American Society of Anesthesiologists issued guidelines recommending preoperative withholding of GLP-1 RAs used for type 2 diabetes management and weight loss before surgical procedures without any change in the current ASA fasting rules [33]. This is the current update in October 2024 after the multi‑society clinical practice guidance by the Surgery for Obesity and Related Diseases, Clinical Gastroenterology and Hepatology and Surgical Endoscopy for safely managing patients needing GLP-1 RAs therapy in type 2 diabetes, overweight and obesity, and heart failure during the periprocedural period [40]. In June 2023, the Canadian Anesthesiologists’ Society published a Medication Safety Bulletin suggesting that anesthesiologists consider patients taking Semaglutide as potentially having a full stomach despite typical fasting periods and, if prolonged preoperative holding is not feasible, aspiration risk reduction strategies should be considered, such as avoidance of deep sedation or general anesthesia, if possible; required use of a rapid sequence induction; and use of gastric ultrasound to help guide decision making [41]. For example, the Brazilian Society of Diabetes suggests a 3-week preoperative discontinuation of GLP-1 RAs before deep sedation and anesthesia [42].

Finally, the latest European Society of Anesthesiology and Intensive Care updated guidelines published in January 2025 have extended the paragraph about GLP-1 RAs and recommended that when a GLP-1 RA is prescribed as a weekly injection for glycemic control, considering the long half-life of GLP-1 RAs, it should be paused at least 1 week before a scheduled procedure requiring sedation/anesthesia [43]. If these drugs are given for obesity, 2 weeks (three half-lives) of suspension are recommended, and whenever possible, a gastric ultrasound should be performed. If the procedure is urgent and postponement is not desirable, endotracheal intubation by rapid sequence induction/intubation is advised. In the case of oral formulation, this last guideline also recommended discontinuing GLP-1 RAs on the day of the procedure. Moreover, a clear fluid diet should be encouraged at least 24 h before any procedure on a patient taking GLP-1 RAs [43]. Of course, all these recommendations by all these Societies should be intended as clinical practice statements (CPS) and suffer a lack of data supporting them as well as a lack of data on the benefits of continuing GLP-1 RAs during the perioperative period, specifically in patients with diabetes mellitus, cardiovascular risks, and renal disease. Although rare at the moment, some groups from Amsterdam with A. H. Hulst and J. A. W. Polderman et al. [44] in agreement with the Association of Anesthetists for day-case surgery say, “Although Anesthesiologists should be aware of the theoretical side effects such as delayed gastric emptying and possible nausea and vomiting, GLP-1-RAs can be considered safe and effective in the perioperative period and recommend continuing all GLP-1-RAs throughout the perioperative period” [45]. They argue that the benefits of perioperative continuation outweigh the risk of withholding these medications, and therefore they propose a no withholding policy for all GLP-1 RAs. We therefore recommend that all GLP-1 RAs be continued during the perioperative period.

### Discussion, proposal of clinical approach based on evidence

As the narrative of perioperative medication use unfolds, it becomes increasingly evident that initiating or discontinuing certain drugs before anesthesia is a delicate and weighty task, demanding the utmost caution and precision in decision-making. The weight of these responsibilities underscores the critical role of anesthesiology as a “perioperativist” in ensuring patient safety [46, 47]. Looking at this literature discord, for anesthesiologists working in everyday practice, is akin to gazing into the future through a crystal ball, which demands the utmost caution and precision because the decisions made in this process carry significant weight and can profoundly impact patient outcomes. For instance, the pivotal role of the Mangano et al. trial in 1996 shaped our understanding of beta-blocker use [48]. This trial, involving over 200 randomized patients treated with atenolol or placebo, demonstrated sustained decreases in postoperative mortality in those who received atenolol, setting a significant precedent in the field to prescribe beta-blockers in high-risk surgical patients before anesthesia. However, the new millennium brought new randomized trials that failed to confirm the previous early study’s promising results and highlighted the potential for beta-blocker harm. The Peri-Operative-Ischemic-Evaluation (POISE) study in 2008, which randomized 8351 patients with preoperative metoprolol vs. placebo showed lower rates of postoperative myocardial infarction but higher rates of stroke and death in high-risk surgical patients who took beta-blockers [49]. This underscores the necessity for careful consideration and individualized decision-making, making healthcare professionals acutely aware of the potential risks and the need for precision in their decisions [43]. This realization led to an immediate downgrade of beta-blocker use in the perioperative period in light of the potential harm associated with their overuse. The perioperative beta-blocker story is a classic example of reversals in recommendations for medical practice [50–54]. Continuation vs. discontinuation of renin-angiotensin system inhibitors before major noncardiac surgery is another important example of the same story [55]. Suppose that the maintenance of arterial pressure will rely more predominantly on the renin-angiotensin or vasopressin axes during anesthesia when the sympathetic tone is inhibited. Taking angiotensin system inhibitors before anesthesia could lead to systemic hypotension, which is very difficult to treat. Again, the literature includes cases of refractory hypotension related to administering these drugs in the perioperative period. It explains the recommendation to withhold them, considering the magnificence of physiologic plausibility in guiding changes in clinical practice [56]. The continuation vs. discontinuation of renin-angiotensin system inhibitors before major noncardiac surgery—The Stop-or-Not Randomized Clinical Trial—showed that in patients undergoing major noncardiac surgery and treated long-term with renin-angiotensin system inhibitors, a continuation strategy of the medication was associated with a similar rate of all-cause mortality and major postoperative complications compared with a discontinuation strategy. This principle, which precedes randomized controlled trial evidence, underscores the paramount importance of scientific rationale in medical decision-making [57].

In 2025, Dixit et al. published a study using an extensive database (Merative MarketScan Commercial Database of about 250 million individuals younger than 65 years enrolled in employer-sponsored health insurance plans) evaluating the risk of postoperative respiratory complications among patients with diabetes and a prescription for a patient under GLP-1 RAs who underwent emergency surgery between January 1, 2015, and December 31, 2021 [58]. This result seems to reply to the ACE-inhibitor story [42]. Of the 3502 (14.8%) patients taking GLP-1 RAs, the incidence of postoperative respiratory complications was 3.5% and 4.0% for those without (odds ratio [OR], 0.85; 95% CI, 0.70–1.04; *p* = 0.12) with no significant difference in the incidence of postoperative respiratory complications between these two groups (adjusted OR, 1.03; 95% CI, 0.82–1.29; *p* = 0.80) [44]. In other words, the perioperative use of GLP-1 RAs in patients undergoing emergency surgery was not associated with a higher risk of postoperative respiratory complications compared with patients not using GLP-1 RAs. Thus, the question becomes, is it possible that GLP-1 RAs and pulmonary aspiration under deep sedation and general anesthesia by case report and supported by gastric ultrasound and EGDS show an association? Should we be prudent until new evidence favors prolonged solid fasting before anesthesia for 24 h? Can we use gastric ultrasound as the only exam in elective situations, being aware that in case of surgical cancellations, this increases patients’ stress and challenges the problem of operating room time notes? Or should we side with the protective effect of GLP-1 RAs, being aware that the aspiration risk can be mitigated through modification of tracheal intubation/extubation techniques and feeling that their benefits do not justify suspicion of GLP-1 RAs in type 2 DM? In that way, could better GLP-1 RAs perioperative glycemic control reduce postoperative infections, stabilize the cardiovascular system, protect the kidney, and reduce systemic inflammation? Or more importantly, could the withholding of GLP-1 RAs result in an adverse “withdrawal” effect? This last resembles the fact that in the study by Managano et al., patients who were taking beta-blockers but were randomized to placebo had had their home beta-blockers discontinued before randomization, potentially creating “withdrawal” effects that might have led to relatively worse outcomes in this group [48]. Furthermore, at the moment, we lack data about glycemic control in stopping GLP-1 RAs in these patients. It is possible that the derangement in glycemic control will likely deteriorate, especially in those with poor control, requiring multidrug regimens, taking insulin, or taking high doses of antihyperglycemics. Moreover, acute hyperglycemia slows gastric emptying, so this may negate the benefit of stopping the drugs before anesthesia [16]. Recently, Klonoff et al. studied over 13,361 adult patients and found that the peri- and postoperative risk complications with or without GLP-1 RAs surrounding the risk of ileus within 7 days, aspiration/pneumonitis, hypoglycemia, and 30-day mortality were not different after surgery with general endotracheal anesthesia [59]. Of course, we are sure that patients who underwent loco-regional or neuraxial procedures should not suspend these drugs. For instance, in a recent study, Magruder et al. used a database called PearlDiver (PearlDiver Technologies, Fort Wayne, IN, USA) from January 1, 2010, to October 31, 2021 to investigate the outcome of patients who underwent total hip arthroplasty; they evaluated 90-day postoperative medical complications, 2-year implant-related complications, 90-day readmissions, in-hospital lengths of stay, and day-of-surgery, as well as 90-day episode of care costs [60]. Not surprisingly, patients taking Semaglutide showed lower rates of readmission within 90 days of surgery (6.2 versus 8.8%; OR 0.68; *p* < 0.01), low rate of prosthetic joint infection (1.6 versus 2.9%; OR 0.56; *P* < 0.01), and deduced the better preoperative glycemic control was probably due to the anti-inflammatory, and immunomodulatory properties of GLP-1RAs [61]. Furthermore, the authors suppose that other infection reductions, like pneumonia, may be due to the effect of GLP-1 RAs mitigating bacterial growth [61]. The American Diabetes Association (Arlington, VA, USA) and the American Association of Clinical Endocrinology (Jacksonville, FL, USA) recommend GLP-1 RAs as first-line drugs along with metformin for patients with type 2 diabetes with an established or high risk of atherosclerotic cardiovascular disease, stroke, transient ischemic attacks, or chronic kidney disease. Semaglutide has been shown to decrease albuminuria in type 2 diabetes mellitus patients with chronic kidney disease [62]. In that way, Vlado Perkovic’s study about Semaglutide in patients with type 2 diabetes and chronic kidney disease among the 3533 participants who underwent randomization (1767 in the Semaglutide group and 1766 in the placebo group) showed a risk of a primary-outcome event was 24% lower in the Semaglutide group than in the placebo group (331 vs. 410 first events; hazard ratio, 0.76; 95% confidence interval [CI], 0.66 to 0.88; *p* = 0.0003) [63]. Additionally, a large clinical trial (“FLOW study”—NCT03819153) was recently terminated early because Semaglutide’s beneficial renal effects had reached statistical significance [64]. Marso et al. randomly assigned 3297 patients with uncontrolled [glycated hemoglobin (HbA1c) 7%] type 2 diabetes mellitus and on two oral antihyperglycemics insulin to receive once weekly Semaglutide (0.5 mg or 1.0 mg) or placebo for 104 weeks and found that the Semaglutide group (SG) had a 26% lower risk of a composite of cardiovascular death, nonfatal myocardial infarction, or nonfatal stroke (*p* < 0.001) regardless of the dose, with a number needed to treat of 45 [65]. All these studies support the emerging benefits of continuing GLP-1 RAs during the perioperative period, specifically in type 2 diabetes mellitus. Furthermore, stopping long-acting GLP-1 RAs before surgery is a challenge. Effective discontinuation would require stopping > 14 days in advance, affecting glycemic control for a similar period. As patients are often seen only shortly before surgery, this policy could lead to unnecessary postponement of surgery.

In this regard, high-quality studies are needed to provide definitive answers about who should suspend these drugs, for example, obese patients due to the fact of difficult intubation, and who should not, for example, type 2 diabetes mellitus for their cardiovascular, renal, and cerebral positive side effects. In other words, it is possible that one size does not fit all. Therefore, anesthesiologists must consider individual patient factors and medical history, the type of GLP-1 RAs used, the dosing regimen (daily vs. weekly), the duration of GLP-1 RAs use, the indication of use (diabetes mellitus vs. weight loss), coexisting medical diseases, and last but not least, the opinion of the patient.

## Conclusion

**Fig. 2:**
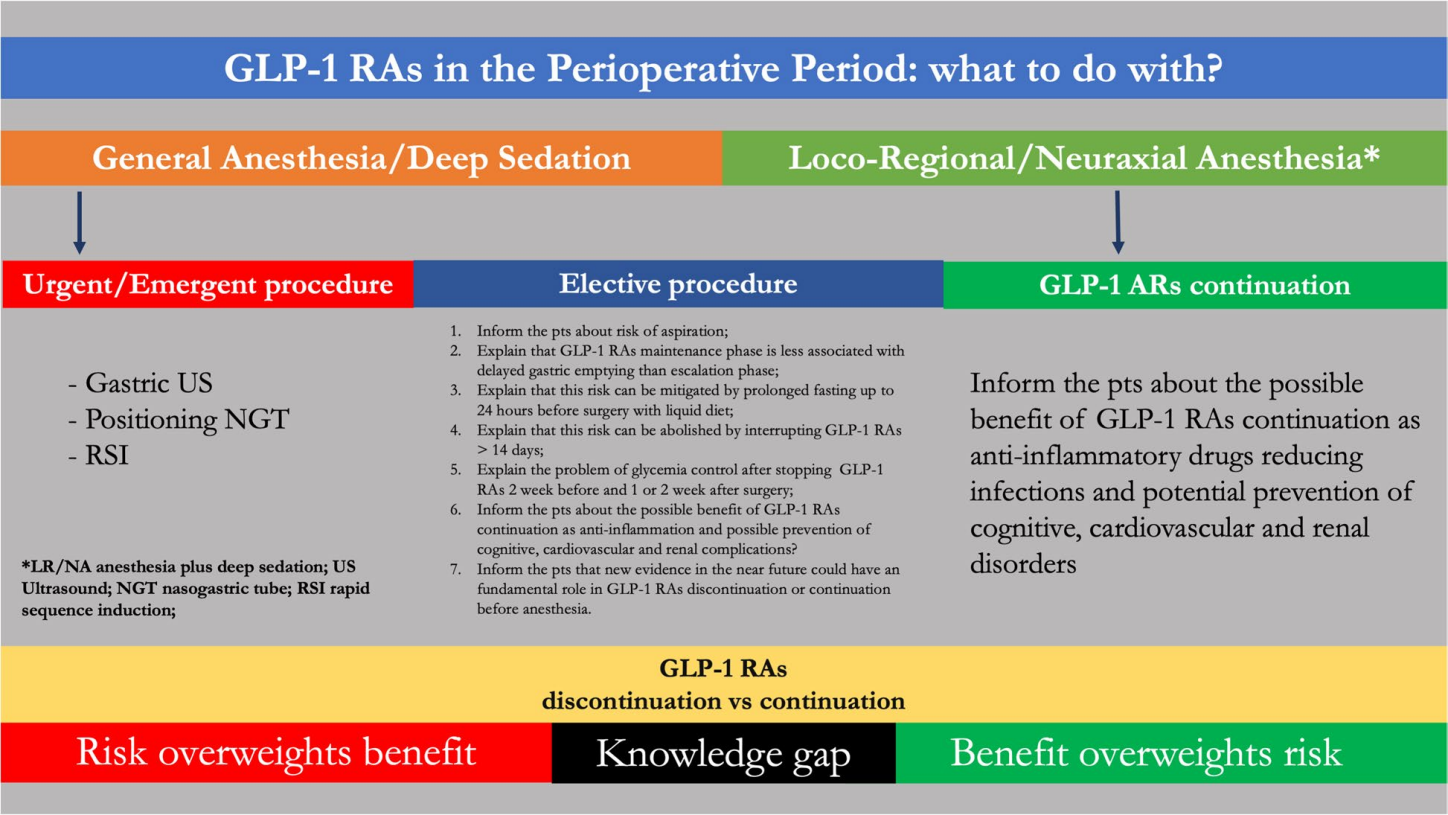
GLP-1 Ars in the perioperative period

Given the variability of the guidelines at the moment, collaboration between anesthesiologists, endocrinologists, and surgeons/proceduralists regarding managing patients on GLP-1 RAs is vital to ensuring safety and quality in the perioperative period. Therefore, the debate about GLP-1 RAs in the perioperative period will probably continue until the next studies are available (Fig. 2).

## Supplementary Information

Supplementary Material 1.

## Data Availability

No datasets were generated or analysed during the current study.

## Abbreviations

GLP1-RAs: Glucagon-like peptide-1 receptor agonists
FDA: Food Drug Administration
EMA: the European Medicines Agency
EGDS: Esophagogastroduodenoscopies
