# Supplementary material for: A narrative review of glucagon-like peptide-1 receptor agonists prior to deep sedation or general anesthesia
Source: J Anesth Analg Crit Care. 2025 Mar 28;5:16. doi: 10.1186/s44158-025-00237-y (PMC11951578; doi:10.1186/s44158-025-00237-y)
Supplement: Supplementary file 1 — Supplementary Material 1. [file 44158_2025_237_MOESM1_ESM.docx]

**Supplemental Material 1**

**Methodologist:** Prof. Daniele Guerino Biasucci

**PICO Framework:**

**Population (P):** Patients taking GLP-1 receptor agonists (e.g., semaglutide, liraglutide, dulaglutide) who are undergoing deep sedation or general anesthesia.

**Intervention (I):** Pausing or discontinuing GLP-1 receptor agonists prior to deep sedation or general anesthesia.

**Comparison (C):** Continuing GLP-1 receptor agonists without pausing prior to deep sedation or general anesthesia.

**Outcome (O):** Implications such as aspiration risk, gastric emptying rates, perioperative complications, or anesthesia-related adverse events.

**PubMED search strategy:**

("GLP-1 receptor agonists" OR "GLP-1 RA" OR "glucagon-like peptide-1 receptor agonists" OR semaglutide OR liraglutide OR dulaglutide OR exenatide OR lixisenatide)

AND ("deep sedation" OR "general anesthesia" OR "perioperative care" OR "anesthesia complications")

AND ("gastric emptying" OR "aspiration risk" OR "perioperative fasting" OR "preoperative management" OR "anesthesia safety")

AND ("pause" OR "discontinue" OR "withhold" OR "stop" OR "interrupt")

**Scopus Search strategy:**

(TITLE-ABS-KEY("GLP-1 receptor agonists" OR "GLP-1 RA" OR "glucagon-like peptide-1 receptor agonists" OR semaglutide OR liraglutide OR dulaglutide OR exenatide OR lixisenatide)

AND TITLE-ABS-KEY("deep sedation" OR "general anesthesia" OR "perioperative care" OR "anesthesia complications")

AND TITLE-ABS-KEY("gastric emptying" OR "aspiration risk" OR "perioperative fasting" OR "preoperative management" OR "anesthesia safety")

AND TITLE-ABS-KEY("pause" OR "discontinue" OR "withhold" OR "stop" OR "interrupt"))

We used filters to limit results to the last 5 years for the most relevant evidence written in English.

We included MeSH terms in PubMed.
